# The Neurobiology of Alcoholism in Genetically Selected Rat Models

**Published:** 1997

**Authors:** Robert B. Stewart, Ting-Kai Li

**Affiliations:** Robert B. Stewart, Ph.D., is an assistant scientist in the Department of Psychology, Purdue School of Science, Indiana University/Purdue University at Indianapolis, Indiana. Ting-Kai Li, M.D., is a distinguished professor of medicine and biochemistry at the Indiana University School of Medicine, Indiana University, Indianapolis, Indiana

**Keywords:** animal strains, selective breeding, AOD preference, amount of AOD use, AOD tolerance, neurotransmitters, reinforcement, drug therapy, literature review

## Abstract

Rats selectively bred for their tendency to drink large or small quantities of alcohol are a useful model for investigators examining the possible neurobiological processes underlying alcoholism. Studies with the alcohol-preferring (P) and alcohol-nonpreferring (NP) and the high-alcohol-drinking (HAD) and low-alcohol-drinking (LAD) pairs of rat lines developed at Indiana University have illustrated differences in several behavioral and neurobiological characteristics associated with alcohol consumption. Specifically, compared with alcohol-avoiding rats, rats with an affinity for alcohol have a greater sensitivity to the stimulatory effects of low to moderate doses and a reduced sensitivity to the negative effects of high doses. Rats that voluntarily drink large quantities of alcohol also acquire tolerance to alcohol’s aversive effects. In addition, these rats differ from their alcohol-avoiding counterparts in the levels of several chemical mediators (i.e., neurotransmitters) found in the brain, including serotonin, dopamine, gamma-aminobutyric acid (GABA), and the endogenous opioids.

Animal models have been critical to many areas of research, including the investigation of the behavioral and neurobiological processes that may underlie alcohol abuse and alcoholism. The use of animals, rather than humans, in research has two advantages: (1) animal models allow a high degree of experimental control not possible with human subjects (i.e., scientists can focus solely on alcohol’s effects without the interference of confounding factors that may accompany alcoholism in humans, such as liver damage, poor nutrition, or psychiatric disturbances) and (2) animal models permit the use of invasive procedures. This article describes the findings of studies on rats that have been specially bred for their tendencies to drink either large or small quantities of alcohol. In particular, the article focuses on characteristics associated with high and low levels of alcohol drinking that have been investigated in the specially bred lines of rats developed at Indiana University.

## Tolerance, Dependence, and Reinforcement

Historically, animal models of alcoholism have been used most extensively to study alcohol tolerance and physical dependence (see Kalant et al. 1971 for a seminal review in this field; see Kalant 1993 and Hoffman and Tabakoff 1996 for recent reviews on the mechanisms of tolerance and dependence). Tolerance to alcohol occurs when, following chronic consumption, higher doses of alcohol must be ingested to achieve a given effect. Consequently, researchers believe that tolerance accounts for increases in the amount of alcohol consumed over time. Physical dependence is indicated by signs of withdrawal resulting from the absence of alcohol in the body when drinking is discontinued. Because alcohol withdrawal symptoms—which range from anxiety, tremors, hypothermia, and sleep disturbances to hallucinations and seizures—are unpleasant (i.e., aversive), researchers hypothesize that physically dependent people drink to avoid or alleviate these symptoms. In fact, the development of tolerance and physical dependence are considered hallmarks of alcoholism.

These two processes, however, cannot account for the initiation of alcohol drinking or explain why relapse occurs in abstinent alcoholics long after the signs of physical dependence have disappeared. Thus, researchers continue to investigate additional behavioral and neurobiological factors that may underlie alcohol use. Recent studies have focused on a process called reinforcement. In behavioral psychology, reinforcement refers to the connection between a behavior and a stimulus whereby the chance of repeated behavior (e.g., alcohol-seeking) is enhanced if the behavior results in obtaining a reinforcing stimulus (e.g., the desirable effects of drinking an alcoholic beverage). The biological basis of alcohol and other drug reinforcement appears to involve the interaction of these substances with specific systems in the brain that regulate “natural” reinforcing and motivated activities such as eating, drinking, and sex (Wise 1980; Koob and Bloom 1988).

## Selective Breeding Programs

Animal studies of the relationship of reinforcement processes to alcoholism initially were hampered by the fact that most laboratory animals, such as rats and mice, will not voluntarily consume alcohol in quantities sufficient to produce significant pharmacological effects (Cicero 1979). To overcome this problem, researchers have found numerous environmental manipulations that increase the rates of alcohol self-administration in laboratory animals (Meisch 1984; Samson et al. 1988). For example, the feeding-induced drinking procedure (Meisch 1976) involves feeding animals such as rats or mice all or part of their daily ration of dry pelleted chow either during or immediately before daily drinking sessions. This feeding results in thirst, and the animals subsequently drink considerable amounts of the fluid made available to them. Initially, the fluid is only water, but alcohol solutions are then presented in gradually increasing concentrations over several sessions. Finally, the food is no longer presented during the drinking sessions, yet the intake of the alcohol solution remains elevated.

A second environmental manipulation is called the sucrose-fading procedure (Samson 1986). In this procedure, animals are first trained to press a lever to access a sweet sucrose solution containing no alcohol. Over the course of several daily drinking sessions, the sucrose concentration is gradually reduced while alcohol is added at increasingly higher concentrations. Finally, the fluid consists of an alcohol solution with no sucrose, and high alcohol intake is maintained.

In addition to such environmental manipulations, genetic manipulation also has been an effective approach to animal studies of alcohol reinforcement, particularly the use of selective breeding programs.^1^ This approach springs directly from the first experiments on rodent alcohol consumption. The oldest and most straightforward method for measuring voluntary alcohol self-administration in rats is to offer a continuous choice between an alcohol solution and water (Richter and Campbell 1940). Although widely used, this so-called two-bottle preference method has been severely criticized, because the average (i.e., mean) daily dose of alcohol consumed by groups of “normal,” or stock, laboratory rats is not high enough to produce significant levels of alcohol in the blood or brain. In other words, stock rats can metabolize alcohol (i.e., break it down and eliminate it from the body) faster than they consume it. If the rate of alcohol consumption does not exceed the rate of alcohol elimination, then the amount of alcohol in the blood and brain can never achieve significant levels. Thus, it is not surprising that stock rats do not display tolerance, physical dependence, or overt intoxication with such low levels of alcohol intake. More important, researchers cannot determine whether the rats consume alcohol for its pharmacologic effects on the central nervous system (CNS) or for other reasons, such as to alleviate hunger or thirst or simply for its taste or smell.

The low mean alcohol intake by stock rats reflects the fact that most rats within a given population avoid alcohol. Much variability exists in the amount of alcohol consumed by individual rats, however. A small percentage of rats within a given population will drink relatively large amounts of alcohol, and a small percentage will drink relatively little. Selective breeding capitalizes on this variation in preference for alcohol over water and has resulted in the development of lines of rats that will consistently self-administer large or small quantities of alcohol when given continuous access to two bottles, one containing a 10-percent alcohol solution and the other containing water alone. Rats bred for their high affinity for alcohol typically consume more than 5 grams of alcohol per kilogram (g/kg) of body weight per day, whereas rats bred for a low affinity for alcohol typically ingest less than 1 g/kg per day. Several pairs of rat lines have been produced through genetic selection for alcohol preference/aversion, including the University of Chile UChA/UChB lines (Mardones and Segovia-Riquelme 1983), the Finnish Alko alcohol-preferring and alcohol-avoiding (AA/ANA) lines (Eriksson 1968), the Sardinian sP/sNP lines (Fadda et al. 1989), and the Indiana University alcohol-preferring and -nonprefer-ring (P/NP) lines and high- and low-alcohol-drinking (HAD/LAD) lines (Lumeng et al. 1995).

**Figure 1:**
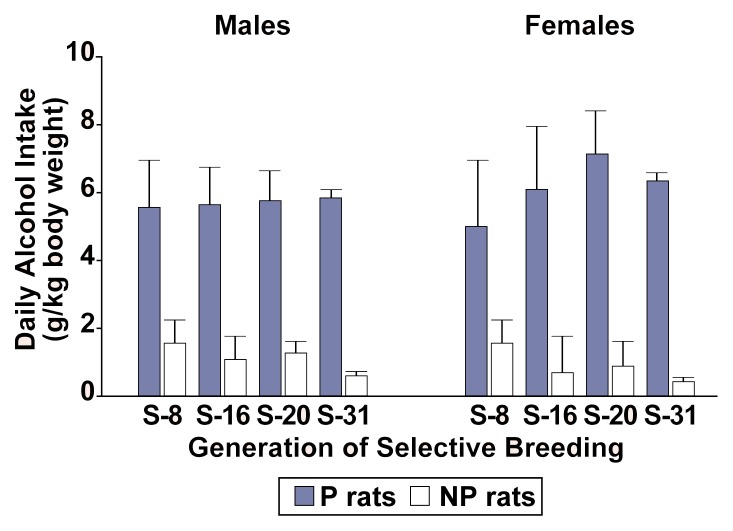
Mean free-choice alcohol consumption (grams of alcohol per kilogram [g/kg] of body weight per day) by recent generations of selectively bred alcohol-preferring (P) and -nonpreferring (NP) rat lines. Males and females of both lines show similar rates of alcohol consumption.

An important criterion for the scientific usefulness of these genetically selected rat lines is maintenance of the preference for (or aversion to) alcohol through successive generations. Figure 1 indicates that in the P/NP rat lines, daily alcohol consumption has been relatively stable since the eighth generation of selective breeding. The P rats, for example, consistently drink more than 5 g/kg per day, resulting in blood alcohol concentrations (BAC’s) of up to 0.2 percent and the development of alcohol tolerance and physical dependence following periods of chronic alcohol self-administration (Li et al. 1988; Li and McBride 1995; Lumeng et al. 1995). Selectively bred rats, such as the P rats, satisfy Cicero’s (1979) rigorous criteria for an animal model of alcoholism (see box, p. 173). In addition, rats selectively bred for alcohol preference add experimental evidence to the importance of genetic factors in determining the risk for alcoholism in humans (Cloninger 1987) and provide an opportunity to determine whether a genetic basis exists for the association between high alcohol consumption and other behavioral and neurobiological characteristics.

**Criteria for an Animal Model of Human Alcoholism**

The criteria for an animal model of human alcoholism are as follows:

- Given a choice between an alcohol solution and another solution (such as water), the animal must voluntarily consume alcohol in an amount sufficient to produce meaningful blood alcohol concentrations (BAC’s). The animal should drink alcohol solely for its pharmacological effects, not for its caloric value or its taste or smell.
- Following a period of chronic alcohol consumption, the animal must develop tolerance, as demonstrated by a reduction in the effects of the same dose of alcohol and the same BAC.
- Following a period of chronic alcohol consumption, the animal must develop alcohol dependence, as demonstrated by behavioral and biological responses characteristic of acute alcohol withdrawal and confirmation of alcohol’s ability to act as a reinforcer (i.e., its ability to increase the chance that alcohol-seeking behavior will occur).

SOURCE: Adapted from Cicero, T.J. A critique of animal analogues of alcoholism. In: Majchrowicz, E., and Noble, E.P., eds. *Biochemistry and Pharmacology of Ethanol.* Vol. 2. New York: Plenum Press, 1979. pp. 534–535.

## Characteristics Associated With High and Low Alcohol Drinking

The following survey of research findings emphasizes studies of the original pair of rat lines selectively bred at Indiana University, the P/NP rats, as well as a second pair of rat lines, the HAD/LAD rat lines, which were developed to replicate and confirm the research findings obtained with the P/NP lines. Unless otherwise noted, the research described was carried out by Li and colleagues at Indiana University. For more specific references, extensive reviews and bibliographies on these and other rat lines are provided by Li and colleagues (1988, 1993), Li and McBride (1995), and Lumeng and colleagues (1995).

### Alcohol-Related Traits

The amount of alcohol that an animal consumes is controlled in part by two competing factors: (1) the reinforcing effects that encourage intake (e.g., euphoria or, conversely, the alleviation of dysphoria or negative emotional states, such as anxiety) and (2) the aversive effects that limit intake (e.g., unpleasant taste, motor-skills impairment, or negative physical reactions, such as dizziness or vomiting). Whether alcohol is reinforcing or aversive depends to some extent on the amount of alcohol intake. Low doses of alcohol generally are reinforcing, but high doses tend to be aversive. Numerous studies have been conducted to characterize the reinforcing and aversive effects of alcohol in the P/NP and HAD/LAD rat lines (see Lumeng et al. 1995 for a review).

**Figure 2:**
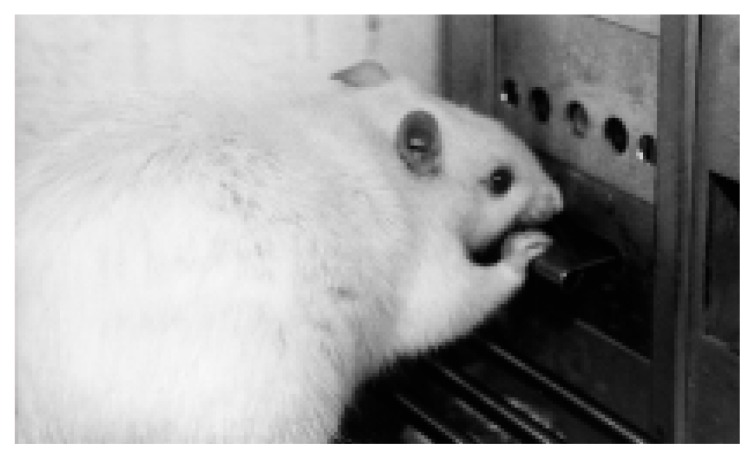
A female alcohol-preferring (P) rat presses a lever in an operant chamber. Each time the lever is pressed, 0.1 milliliter of alcohol solution is delivered into a well to the right of the lever. P rats work to obtain alcohol solutions at concentrations as high as 40 percent (the typical concentration of unmixed hard liquors, such as straight whiskeys). Photograph by Maggie Johann Stewart

Although differences in the rats’ alcohol intake in the two-bottle preference test suggest that alcohol is more reinforcing for P and HAD than for NP and LAD rats, recent studies provide additional evidence for this conclusion. Rats can be trained to press a lever or perform some other work to obtain alcohol (see figure 2), a paradigm known as operant responding, and the alcohol “reward” received as a result of the rats’ correct action can increase the frequency of the operant response (i.e., it is reinforcing). In both P and HAD rats, operant responding is maintained by the delivery of alcohol over a wide range of experimental conditions. For example, the period of daily alcohol availability may be limited to, say, 30 minutes (Schwarz-Stevens et al. 1991) or it may be continuous (Files et al. 1993). Several methods of initiating the operant response for alcohol have been investigated, including the feeding-induced drinking and sucrose-fading procedures (Schwarz-Stevens et al. 1991; Ritz et al. 1994). Alcohol concentrations as low as 1 percent and as high as 40 percent maintain responding in P and HAD rats. In contrast, responding by NP and LAD rats under the same conditions either is not maintained or is much lower than responding by their alcohol-preferring counterparts.

Alcohol also functions as a reinforcer by nonoral routes of administration for P, but not NP, rats. For example, P rats will learn the correct operant response that results in alcohol administration through a tube directly into the stomach (i.e., intragastric administration). Conversely, stock rats will engage in intragastric alcohol self-administration only after developing physical dependence during a period of forced alcohol infusion. P rats, but not NP rats, also will learn to perform the correct response that administers alcohol through a tube directly into the ventral tegmental area of the brain, a region implicated in reinforcement by alcohol and other drugs. By bypassing the mouth, these methods of alcohol consumption eliminate all of the behaviors and stimuli (e.g., taste) associated with oral consumption as factors that may explain the line differences in the reinforcing effects of alcohol.

Increases in spontaneous motor activity^2^ and other signs of behavioral arousal following drug administration are strongly associated with the reinforcing effects of many drug classes, including stimulants, opiates, and sedative-hypnotics. Interestingly, low doses of alcohol also produce increases in spontaneous motor activity shortly after alcohol administration in P and HAD rats, but not in NP and LAD rats. In addition, studies using techniques such as electroencephalography (EEG), a method of measuring brain electrical activity, also provide evidence that alcohol produces more arousal in P than in NP rats.

Because alcohol usually is consumed orally, it is of interest to determine whether P and HAD rats differ from NP and LAD rats in their reactions to alcohol’s flavor (i.e., taste reactivity). A variance in avidity for alcohol may result from different preferences for the taste of alcohol solutions, for example. To test the taste reactivity of the selectively bred rats, Kiefer and coworkers (Bice and Kiefer 1990; Kiefer et al. 1995) placed drops of alcohol solution into the mouths of rats who had never previously consumed alcohol (i.e., alcohol-naive rats) and noted their facial responses, which were then quantified to measure how much the rats liked or disliked the flavor of the solution. The investigators did not find any differences in taste reactivity between the alcohol-preferring (P and HAD) and -nonpreferring (NP and LAD) rat lines on initial exposure. Next, the researchers gave the rats a two-bottle preference test with alcohol solution and water for 3 weeks, and predictably, the P and HAD rats consistently drank more alcohol than did the NP and LAD rats. Following the two-bottle preference test, a second taste-reactivity test was given, and results indicated that alcohol had become more palatable to the P and HAD rats (although not to the NP and LAD rats) during the period of oral alcohol consumption. The alcohol-preferring rats maintained this increase in alcohol palatability even after 1 month of alcohol abstinence. Thus, preference for the taste of alcohol is not an inherited characteristic in the alcohol-preferring rat lines; instead, this taste preference is acquired through alcohol-drinking experience.

Some of the CNS effects of alcohol, especially at high doses, are aversive or dysphoric. Rats selectively bred for high and low alcohol preference have been tested for their sensitivity to these negative effects. Among the most useful testing methods is the conditioned taste aversion procedure, wherein an animal receives a large, presumably aversive, dose of alcohol by injection at approximately the same time that it receives a particular food or other taste stimulus. In normal rats, pairing a taste stimulus and an aversive dose of alcohol will cause the rat to avoid that taste in the future. Similarly, the conditioned place aversion procedure creates aversion to a particular location by placing the rat there as it experiences the aversive effects of an alcohol injection. In both types of studies, P rats are less sensitive than NP rats to alcohol’s aversive effects.

**Figure 3:**
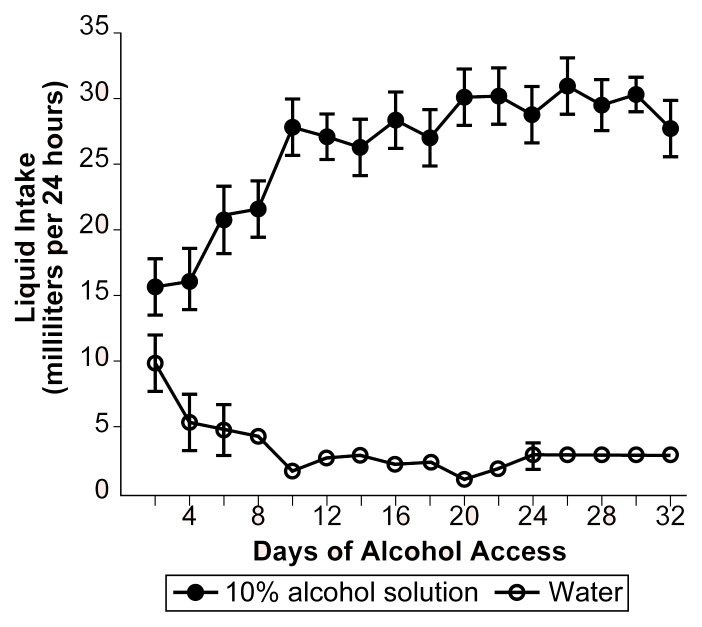
Volume of water (open circles) and concurrently available 10-percent alcohol solution (closed circles) consumed by 18 alcohol-preferring rats during 32 days of chronic alcohol drinking. Data shown are averages for consecutive 2-day periods. The increase in alcohol intake over successive days in these rats is consistent with the development of tolerance.

Furthermore, P rats with histories of oral alcohol consumption, compared with alcohol-naive rats, experience less motor-impairing and aversive effects from high doses of alcohol, suggesting that they developed tolerance following chronic drinking. For example, in one study, P rats were given continuous access to an alcohol solution and water for 32 days. During this period, the rats increased their alcohol consumption by about 50 percent (see figure 3), indicating the development of tolerance. Following the period of oral alcohol self-administration, these rats, along with alcohol-naive P rats serving as control subjects, underwent conditioned taste aversion trials in which they drank a sweetened fluid they had never previously tasted, then immediately received an injection of alcohol. The injected doses were sufficiently high to produce conditioned aversive effects, as indicated by the rats’ avoidance of the sweetened fluid upon subsequent exposure. The alcohol-exposed P rats, however, exhibited an attenuated conditioned taste aversion relative to the alcohol-naive P control rats. Thus, P rats developed tolerance to alcohol’s aversive CNS effects just as they had developed a tolerance for alcohol’s flavor in the taste reactivity tests. This tolerance to aversive CNS effects could contribute, at least in part, to the rats’ high alcohol intake.

In addition to the rats’ acquired reduction in sensitivity to the CNS effects of alcohol (termed “neuronal” or “functional” tolerance), prolonged periods of alcohol self-administration also increase the rate of alcohol metabolism in the liver (termed “metabolic” tolerance) in P rats. Rats of the NP line do not self-administer sufficient quantities of alcohol to develop metabolic tolerance. A comparison of alcohol-naive P and NP rats, however, found no differences in the rates at which alcohol is metabolized in the liver and eliminated from the body. Consequently, when the same amount of alcohol is administered to P and NP rats, both lines achieve the same BAC levels. Thus, the divergent drinking levels and reactions to alcohol seen in P and NP rats apparently are not attributable to differences in alcohol metabolism or elimination, but to differences in neuronal sensitivity to alcohol.

A series of studies has shown that P rats can develop acute tolerance to a single sedative-hypnotic dose of alcohol more quickly and/or to a greater extent than NP rats. That is, P rats recover more quickly than NP rats on a number of tests measuring the depressant effects of alcohol, including motor impairment, lowered body temperature, and regain of righting reflex (i.e., sleep time). Using a behavioral measure of alcohol-induced motor impairment, researchers observed that the tolerance P rats develop to a single dose of alcohol can persist for as long as 10 days, whereas such tolerance in NP rats, which is weaker in the first place, dissipates within 3 days. Differences in initial sensitivity and acute tolerance also have been found in other alcohol-preferring and -nonpreferring rodent lines and strains, such as the AA/ANA rat lines (Le and Kiianmaa 1988) and the C57BL/DBA mouse strains (Tabakoff and Ritzmann 1979). This finding indicates a strong association between tolerance and high voluntary alcohol consumption.

### Traits Not Directly Related to Alcohol

In addition to alcohol-related traits, researchers are interested in detecting other behavioral and biological differences between rats genetically selected for high or low alcohol preference, with the hope that such differences may provide further clues to the genesis and perpetuation of alcoholism. Thus far, however, relatively few studies of this type have been conducted.

Among existing studies, results indicate that P rats exhibit higher spontaneous motor activity than do NP rats when placed in a new environment, but no difference appears between the lines when the environment is no longer novel. These observations concur with the high novelty-seeking personality characteristic that is noted in certain types of human alcoholics (Cloninger 1987). P rats also seem to be more anxious than NP rats on a number of behavioral tests of anxiety, which accords with the notion that alcohol may be self-administered to relieve tension and anxiety.

In addition, P rats exhibit a higher preference than NP rats for oral consumption of highly palatable, nondrug solutions, such as sucrose or saccharin, but intake of plain water and of sour and bitter-flavored solutions does not differ between P and NP rats. Because a preference for sweets highly correlates with high alcohol intake in numerous rodent lines and strains, investigators have suggested that the same, or overlapping, brain mechanisms may be involved in the reinforcement mediated by some drugs (e.g., alcohol) and other palatable substances (e.g., chocolate).

## Neurobiological Differences Associated With High and Low Alcohol Drinking

Biological studies of the P/NP and HAD/LAD rat lines have focused on the study of chemical mediators known as neuro-transmitters^3^ (i.e., neurochemistry) and the identification and mapping of groups of neurons that seem to have similar functions (i.e., neuroanatomy). Neurochemical studies implicate a subset of neurotransmitters with special roles in the control of alcohol-seeking behavior: 5-hydroxytryptamine (5-HT), also called serotonin; dopamine; gamma-aminobutyric acid (GABA); and the body’s own opiatelike substances, the endogenous opioids (i.e., endorphins).

The 5-HT system appears to be a key component in the regulation of food consumption and mood as well as the development of alcohol tolerance. In addition, 5-HT modulates the release of dopamine, thereby directly affecting the dopamine system. In turn, the dopamine system plays a major role in motor activity, drug reinforcement, and the motivation to engage in several other behaviors that may be considered reinforcing or rewarding, such as eating and sex. GABA differs from 5-HT and dopamine in that it is not confined to certain neurons and pathways forming a system. Rather, it is found throughout the brain, conveying inhibitory signals and perhaps interacting with dopamine and other neurotransmitter systems in alcohol reinforcement. Endogenous opioids also act as inhibitory neurotransmitters and are released in response to stresses such as injury, childbirth, and vigorous exercise. In addition, opioids play a role in eating and drinking behaviors and, like GABA, appear to interact with dopamine and other neurotransmitter systems involved in alcohol reinforcement.

Several brain structures appear to participate in a postulated brain “reward pathway,” including the ventral tegmental area, raphe nuclei, lateral hypothalamus, olfactory tubercle, nucleus accumbens, and medial prefrontal cortex and other limbic areas (see glossary, pp. 177–179). The function of this neural pathway system is to regulate behaviors motivated by “natural” rewards such as food, water, and sex. Scientists believe, however, that alcohol and other drugs of abuse (e.g., cocaine and morphine) function as reinforcers by imitating, facilitating, or sometimes blocking the various neurotransmitters involved in this system. Based on neuropharmacological studies and on studies in which rats learn an operant response to electrically self-stimulate their brain “reward” areas, the neurotransmitters 5-HT, dopamine, GABA, and the endogenous opioids all have been implicated in the circuitries of the brain reward pathway (Wise 1980; Koob and Bloom 1988).

One of the most consistent neurochemical and neuroanatomical findings observed in P/NP and HAD/LAD rats is a deficiency of 5-HT in the alcohol-preferring lines (McBride et al. 1991; Li and McBride 1995). Compared with rats that drink little alcohol, the levels of 5-HT in rats that drink large amounts of alcohol are significantly reduced in several brain regions, including the frontal cortex, hippocampus, corpus striatum, thalamus, hypothalamus, pons-medulla, and nucleus accumbens. These regions are involved either in the brain reward pathway or in neural processes that are relevant to alcohol-seeking behavior (e.g., learning, memory, and tolerance development processes). Closer examination of the neurons in some of these brain regions (such as the frontal cortex, nucleus accumbens, and hippocampus) suggests that these differences may be caused by a relative scarcity of 5-HT–containing axons. Interestingly, research has shown that the decrease in 5-HT–containing axons in P rats results in compensatory up-regulation of 5-HT receptor activity (McBride et al. 1991; Li and McBride 1995). That is, the number or sensitivity of the target 5-HT receptors apparently increases to make up for the reduction in the availability of 5-HT.

An abnormality in one of the major components of the brain reward circuitry—the ventral tegmental area-nucleus accumbens dopamine system—also has been associated with high alcohol preference. Specifically, scientists have observed low levels of dopamine and chemicals associated with its breakdown in the nucleus accumbens and anterior striatum of P and HAD rats in the absence of alcohol. This finding is of interest, because abused drugs from many pharmacological classes (including stimulants, opiates, and sedatives), as well as low doses of alcohol, stimulate the release of dopamine in the nucleus accumbens. The P rats may be particularly sensitive to this alcohol-induced dopamine release in the nucleus accumbens. That is, alcohol consumption by P rats may be, in a sense, a regulatory action aimed at increasing the rats’ abnormally low levels of dopamine in the accumbens. Because the 5-HT system plays a role in regulating the dopamine system in the brain reward pathway, the decreased 5-HT innervation noted in alcohol-preferring rat lines also may affect the function of the dopamine system in these rats.

In addition to stimulating dopamine release in the nucleus accumbens, alcohol’s actions on neuronal activity also stimulate GABA receptors. Noting that anxiety-reducing drugs such as the benzodiazepines (e.g., Valium^®^) produce their effects by facilitating nerve signal transmission at synapses using the neurotransmitter GABA, researchers theorize that alcohol may produce its rewarding and anxiety-reducing effects via GABA neurons as well. Interestingly, studies in the P/NP and HAD/LAD rat lines have demonstrated a higher density of axon terminals containing GABA in the accumbens of the rats with high alcohol preference. This suggests a potential for increased GABA activity in P and HAD rats in an area of the brain involved in alcohol reinforcement.

The endogenous opioid systems also are involved in the regulation of alcohol drinking, as evidenced by the ability of opiate drugs to alter alcohol consumption. The endogenous opioid systems have been studied in P/NP rats (Froehlich and Li 1993) and in the Finnish AA/ANA lines (Nylander et al. 1994). The high- and low-alcohol-drinking rat lines differ in opioid activity in the absence of alcohol as well as in alcohol-stimulated opioid activity in the nucleus accumbens and pituitary gland (Froehlich and Li 1993; Nylander et al. 1994). However, the brain reward pathway of the Finnish AA rats does not appear to have low levels of dopamine or 5-HT (Korpi et al. 1988) as is the case with the selectively bred P and HAD rats and other rodent strains that consume large amounts of alcohol (see Li and McBride 1995 for a review).

The apparently discordant findings from the comparison of P/NP and AA/ANA rat pairs may result from variances in the foundation stocks from which the two pairs of genetically selected lines were derived. If so, selective-breeding experiments may achieve the same endpoints (e.g., high and low alcohol preference) by altering different brain mechanisms. Such findings may reflect the multiplicity of mechanisms that may contribute to high alcohol intake. Indeed, the challenge to alcohol researchers and clinicians is that alcoholism results from the interaction of many biological factors (inherited and environmental) and is not a unitary phenomenon.

## Significance

Studies with rats selectively bred for alcohol preference or nonpreference support several hypotheses on factors that may be associated with alcoholism in humans. For example, the demonstrated sensitivity of alcohol-preferring rats to the stimulatory effects of low to moderate alcohol doses is in agreement with the contention that these effects are important in the initiation and maintenance of alcohol drinking. The alcohol-preferring rats also show an innate insensitivity to the aversive effects of alcohol at high doses, which may tend to limit the amount of alcohol consumed by rats that are normal or genetically selected for low alcohol preference. Tolerance development to these aversive effects, which occurs to a greater extent in the alcohol-preferring rats, also may encourage increased alcohol intake. Furthermore, behavioral research with selectively bred rat lines indicates that individual differences in responsiveness to alcohol can be heritable traits and that these animal models are valuable tools for investigating neural mechanisms relevant to alcohol-seeking behavior.

These animal models also have provided the underpinning for a new direction in the treatment of alcoholism and alcohol abuse: testing pharmacotherapies related to the postulated neural reward mechanisms. The 5-HT and opioid systems have long been implicated as having a role in alcohol drinking (Myers and Melchior 1977; Altshuler et al. 1980), and basic research findings, such as the line differences in the brain reward systems of selectively bred rats, have provided a theoretical basis for clinical studies on the effects of drugs that may influence activity in these systems. Various agents that alter 5-HT, dopamine, GABA, and opioid functioning decrease alcohol consumption in animal models, including selectively bred alcohol-preferring rats (see Lumeng et al. 1995 for a review). For example, fluoxetine (Prozac^®^) is an antidepressant drug that inhibits the reuptake of 5-HT by the neurons that secrete it and thereby facilitates 5-HT activity. Fluoxetine has been found to significantly reduce alcohol intake in populations of heavy drinkers (Naranjo et al. 1986), but clinical trials to date have not shown fluoxetine to be effective in treating alcoholism (Litten et al. 1996). Nevertheless, some alcoholic subtypes, such as those with comorbid depression, may respond favorably to fluoxetine. The opioid blocker naltrexone (ReVia^™^ or Trexan^®^) also has been tested in clinical trials with alcoholics (O’Malley et al. 1992; Volpicelli et al. 1992). Subjects receiving naltrexone showed decreases in the mean number of drinking days per week, frequency of relapse, desire to drink (i.e., craving), and the alcohol-induced subjective “high.” Such results suggest that these pharmacological manipulations decrease alcohol’s reinforcing effects. The fact that drugs such as fluoxetine and naltrexone reduce alcohol intake in both rodents and humans supports the predictive validity of the use of genetic animal models for evaluating therapies that can potentially reduce or prevent excessive alcohol consumption.

## Footnotes

1 In selective breeding programs, animals from a genetically heterogeneous population with a certain desired characteristic (e.g., a tendency to drink alcohol) are mated and their offspring are screened for that characteristic. Those offspring bearing the desired characteristic are then bred. If the characteristic is a heritable trait, the process of selection is repeated in successive generations to produce lines of animals that “breed true” for the characteristic.

2 “Spontaneous motor activity” describes the measured amount of movement and exploration that takes place when rats are placed in an enclosure consisting of an open area or “field” surrounded by four walls.

3 For definition of this and other technical terms used in this article, see central glossary, pp. 177–179
